# Supplementary material for: Analysis of statistical correlations between properties of adaptive walks in fitness landscapes
Source: R Soc Open Sci. 2020 Jan 29;7(1):192118. doi: 10.1098/rsos.192118 (PMC7029893; doi:10.1098/rsos.192118)
Supplement: Electronic Supplementary Material from Analysis of statistical correlations between properties of adaptive walks in fitness landscapes [file rsos192118supp1.pdf]

Electronic Supplementary Material from Analysis of statistical correlations between properties of adaptive walks in fitness landscapes

Sandro M. Reia

Instituto de Física de São Carlos, Universidade de São Paulo,

Caixa Postal 369, 13560-970 São Carlos, São Paulo.

Paulo R. A. Campos*

Evolutionary Dynamics Lab,

Physics Department,

Federal University of Pernambuco

Abstract

This electronic supplementary material provides additional figures of the mutational pathways for the Gb1 and NK fitness landscapes, as well as additional correlation tables.

* paulo.acampos@ufpe.br

I. GB1 FITNESS LANDSCAPE - MUTATIONAL PATHWAYS

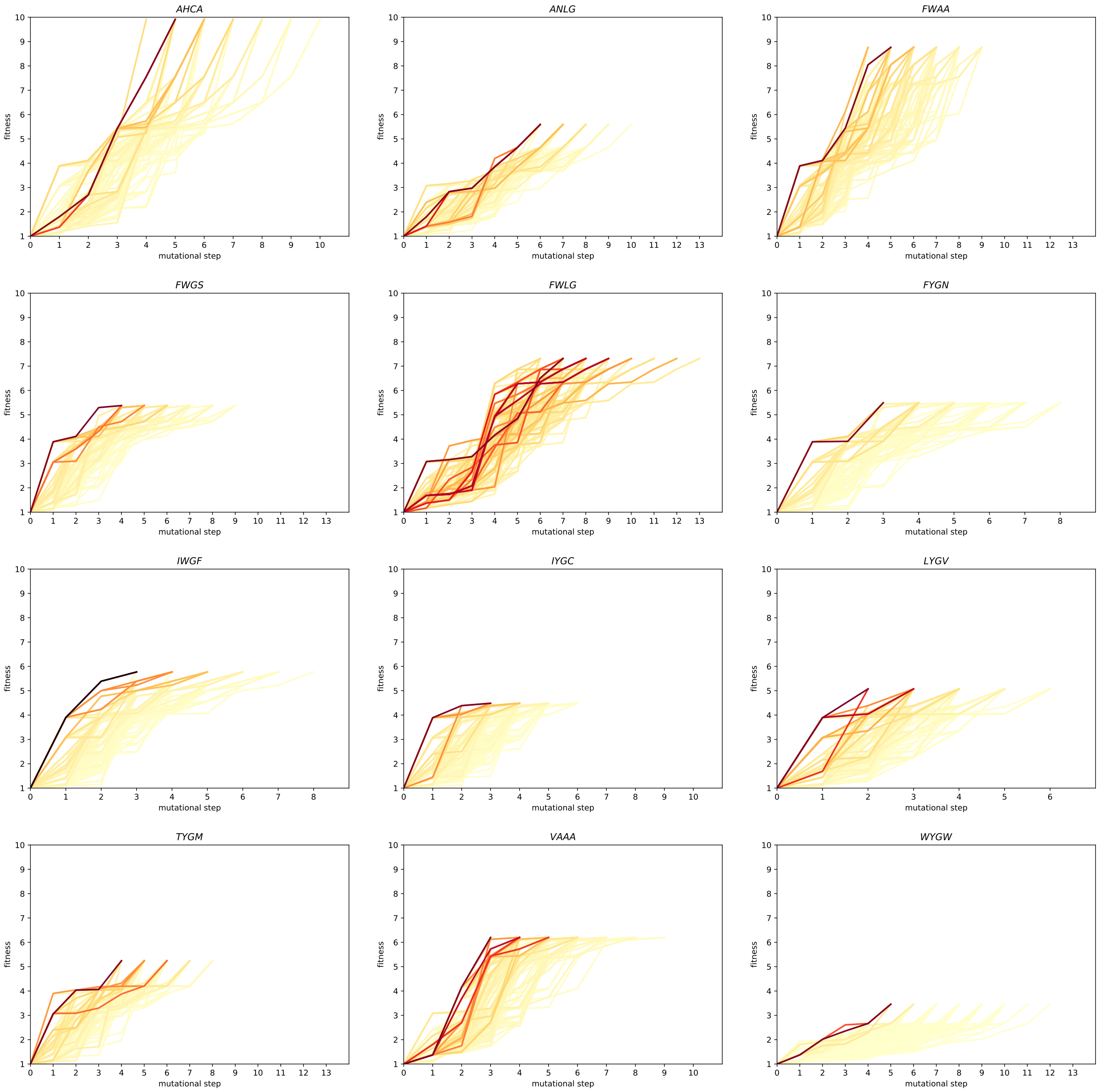

FIG. 1. The set of the 50 most used monotonic mutational pathways to the twelve local maxima of the GB1 fitness landscape that are accessible from the wildtype sequence (VDGV). The corresponding target sequences are AHCA, ANLG, FWAA, FWGS, FWLG, FYGN, IWGF, IYGC, LYGV, TYGM, VAAA and WYGW. AHCA is the global optimum of the GB1 fitness landscape. A color gradient has been used to denote the frequency at which the paths are assessed through the dynamics. The paths were obtained under the probabilistic dynamics. The data of the Gb1 fitness landscape were obtained from Wu et al. [1].

II. NK MODEL - MUTATIONAL PATHWAYS

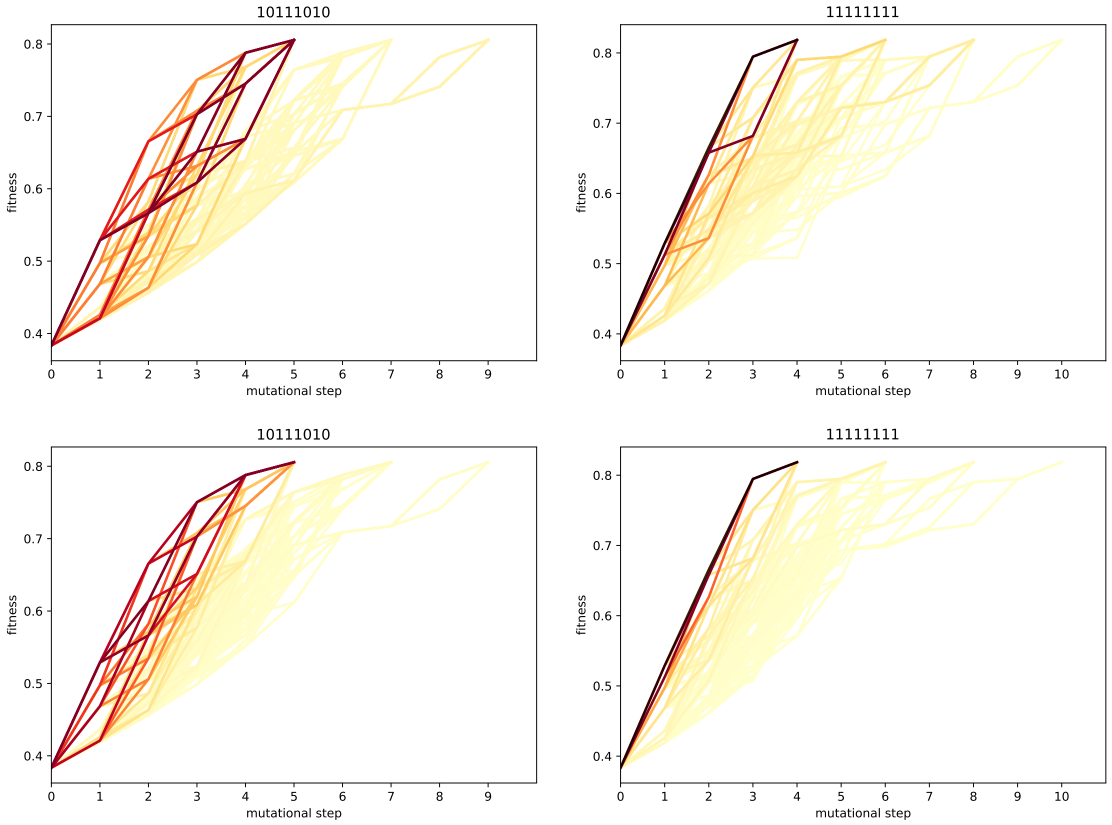

FIG. 2. The set of the monotonic mutational pathways to the two local maxima of a single instance of the NK fitness landscape, with $K = 1$. Upper panels: random adaptive walks. Lower panels: probabilistic adaptive walks.

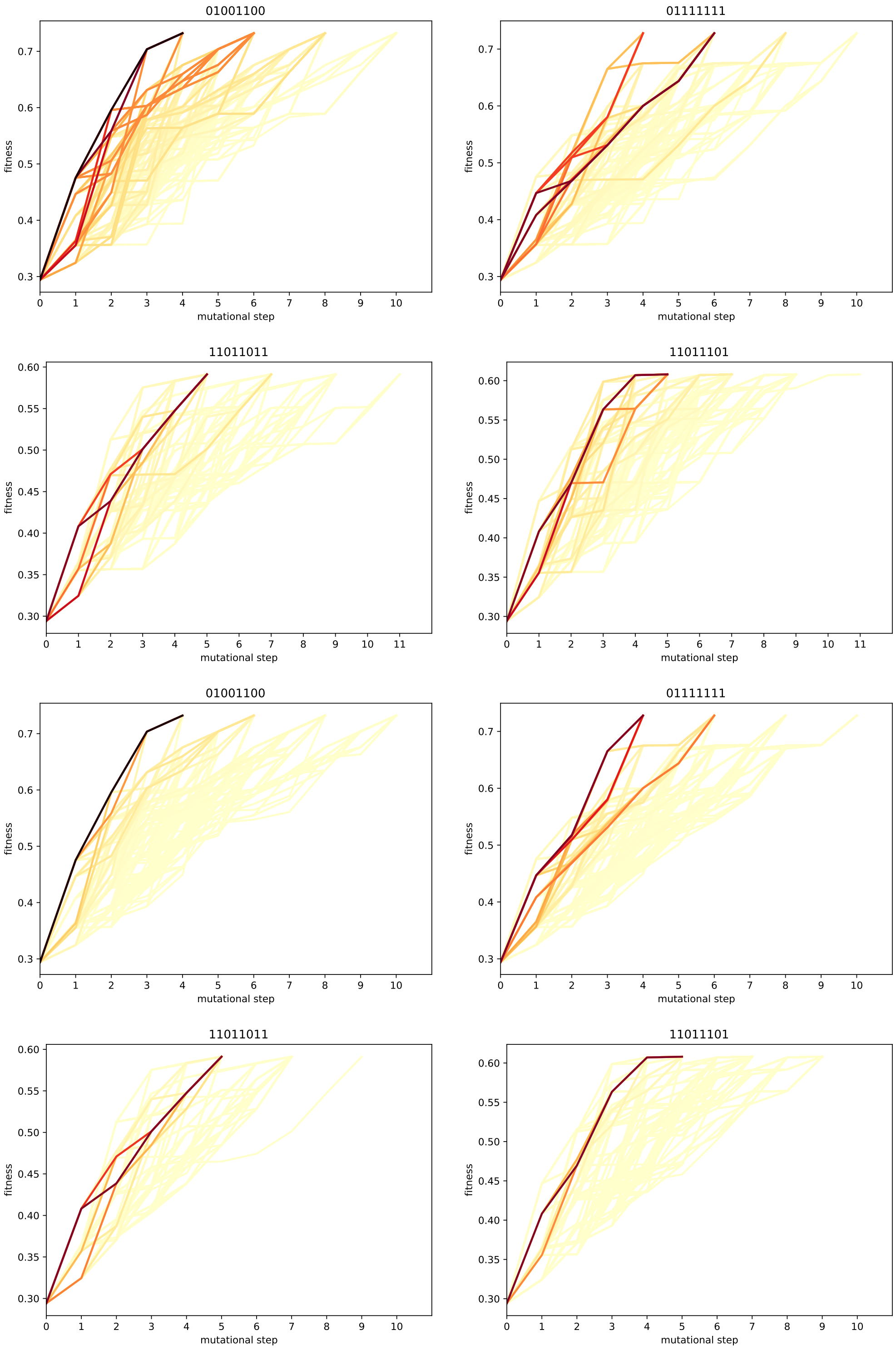

FIG. 3. The set of the monotonic mutational pathways to the two local maxima of a single instance of the NK fitness landscape, with $K = 2$. Upper panels: random adaptive walks (first two rows). Lower panels: probabilistic adaptive walks (last two rows).

III. CORRELATION TABLES - RANDOM ADAPTIVE WALKS

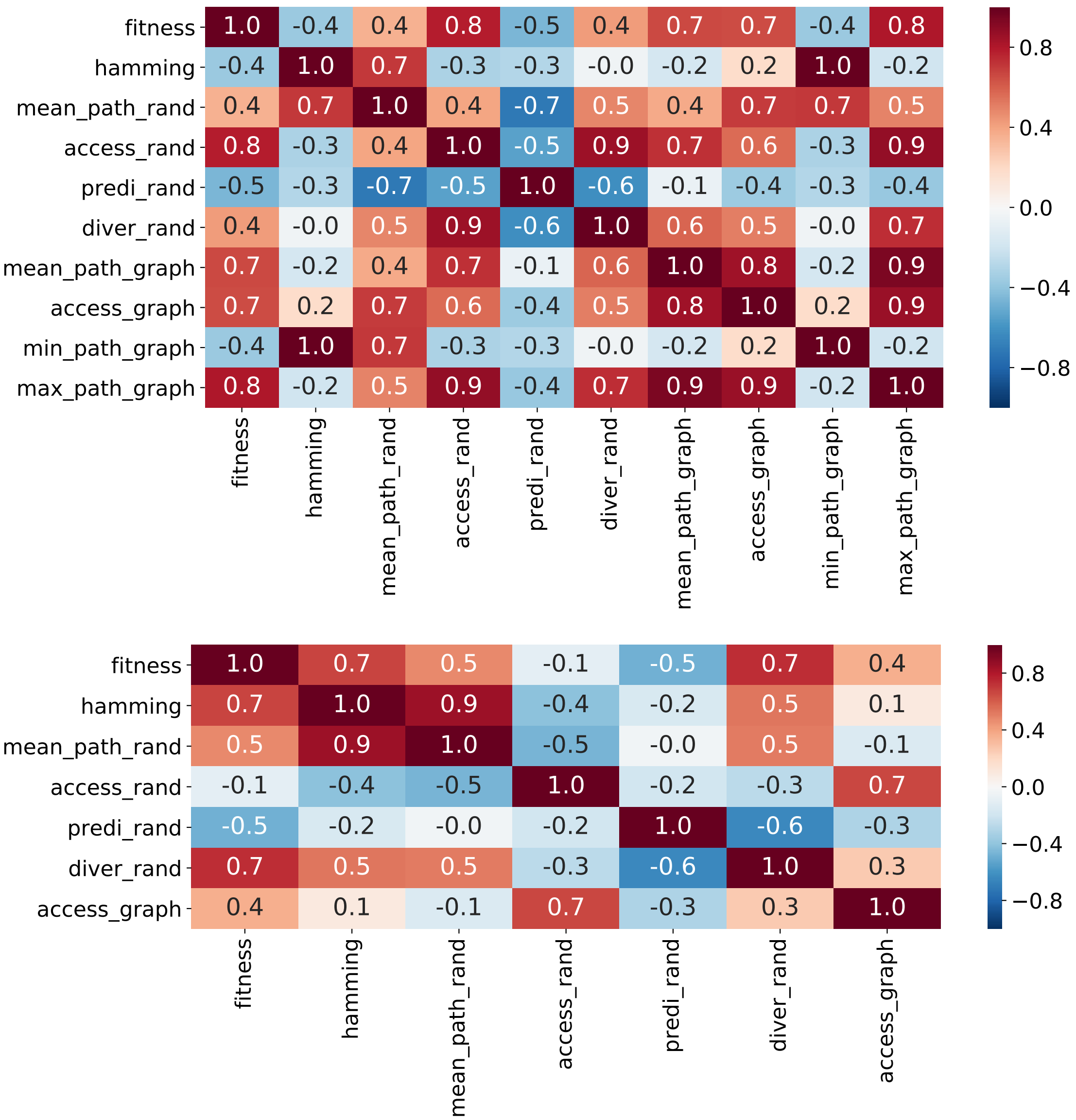

FIG. 4. Correlation tables specifying the level of correlation between random adaptive walks and graph measures of the empirical landscapes Hsp90 in the upper panel and the Gb1 in the lower panel.

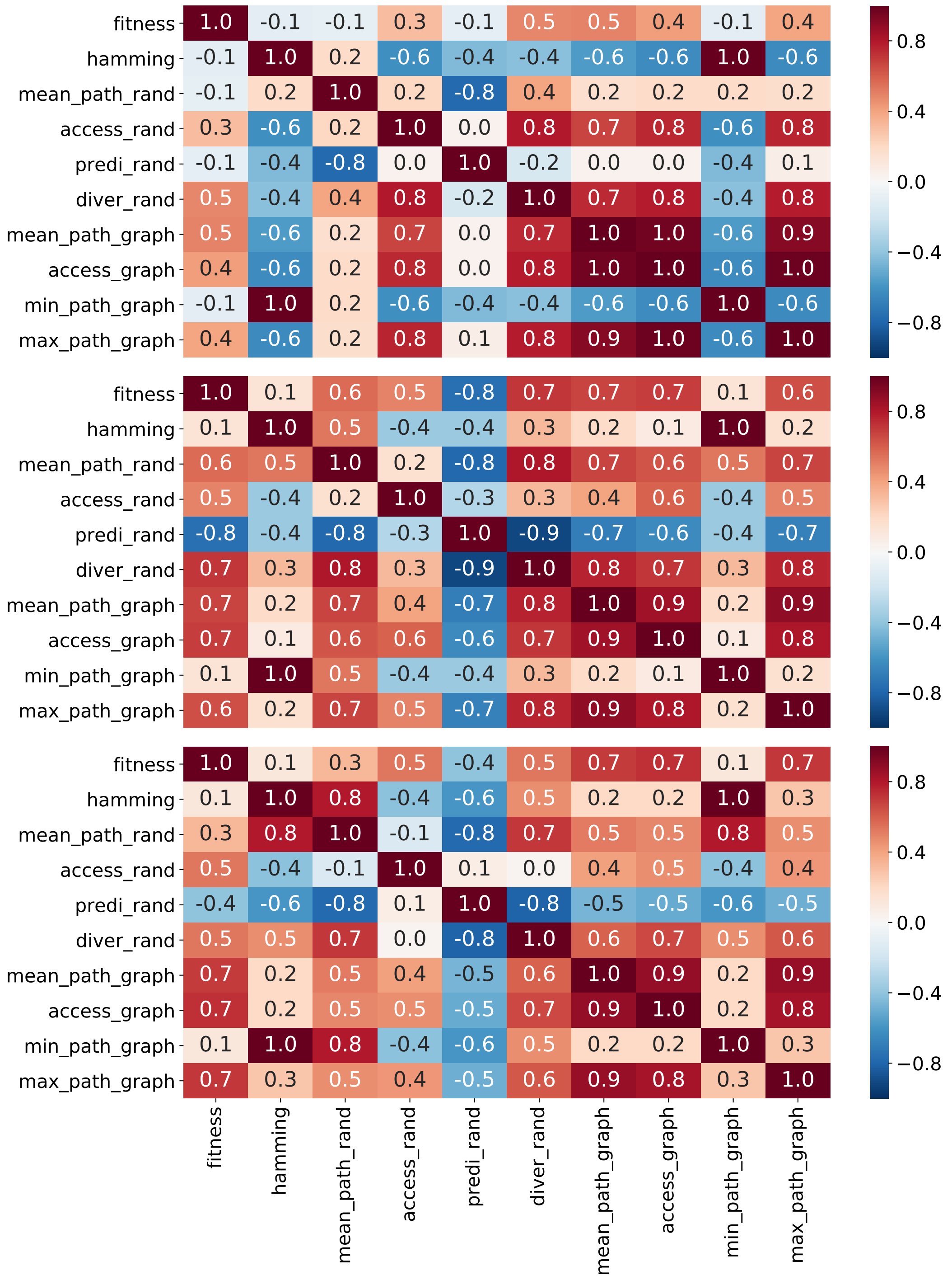

FIG. 5. Correlation between random adaptive walks and graph measures of the NK-model with $N = 8$ and epistasis degrees of $K = 1$ (upper panel), $K = 2$ (middle panel) and $K = 3$ (lower panel).

IV. CORRELATION MATRIX - NK LANDSCAPES

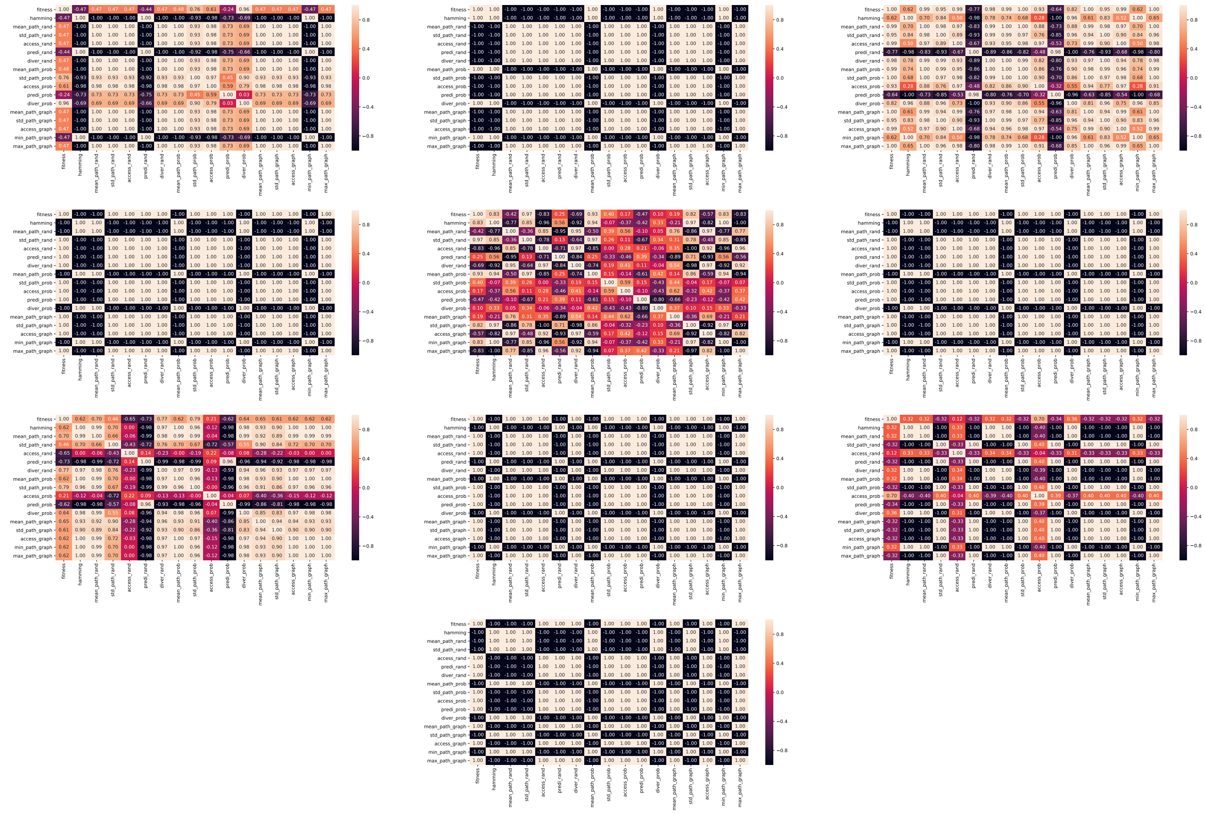

FIG. 6. Correlation tables specifying the level of correlation between pair of variables for the NK fitness landscape. The abbreviations of the variables in the label are declared in Sec. 2.3. The parameter values are $N = 8$ and $K = 1$.

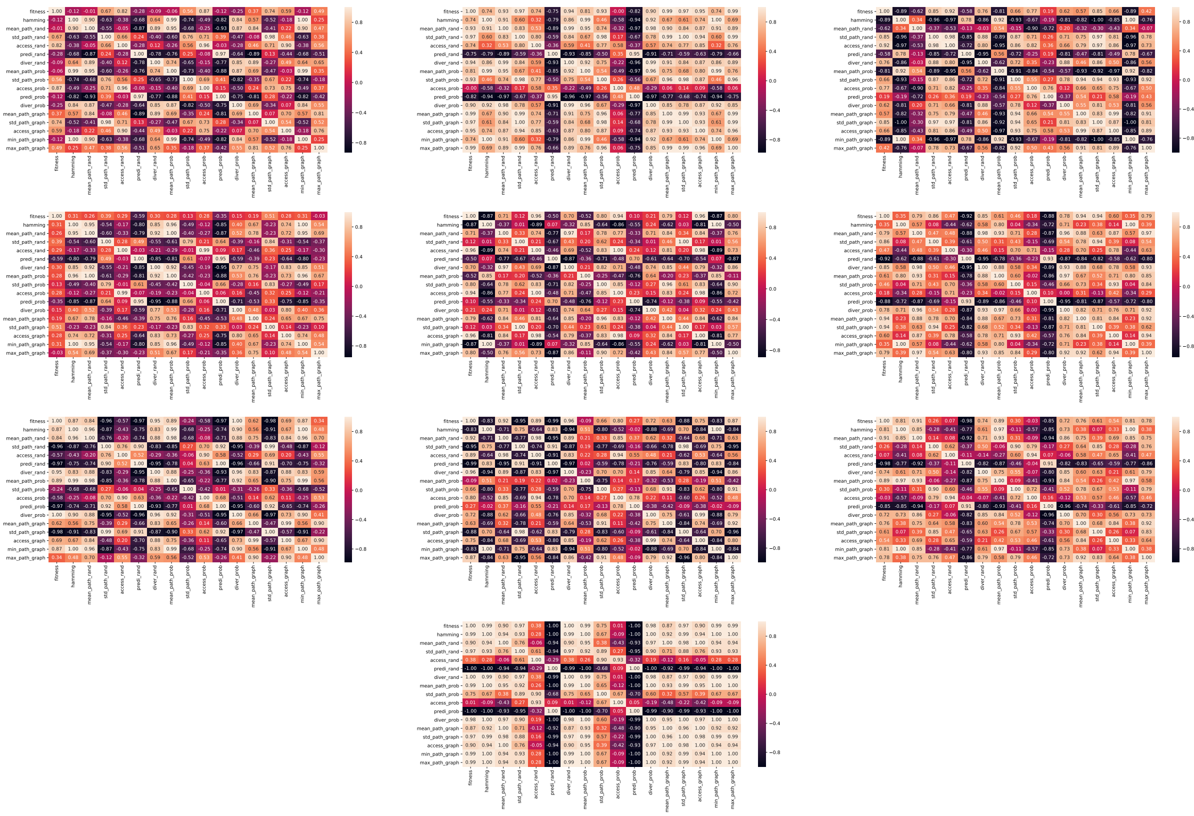

FIG. 7. Correlation tables specifying the level of correlation between pair of variables for the NK fitness landscape. The abbreviations of the variables in the label are declared in Sec. 2.3. The parameter values are $N = 8$ and $K = 2$.

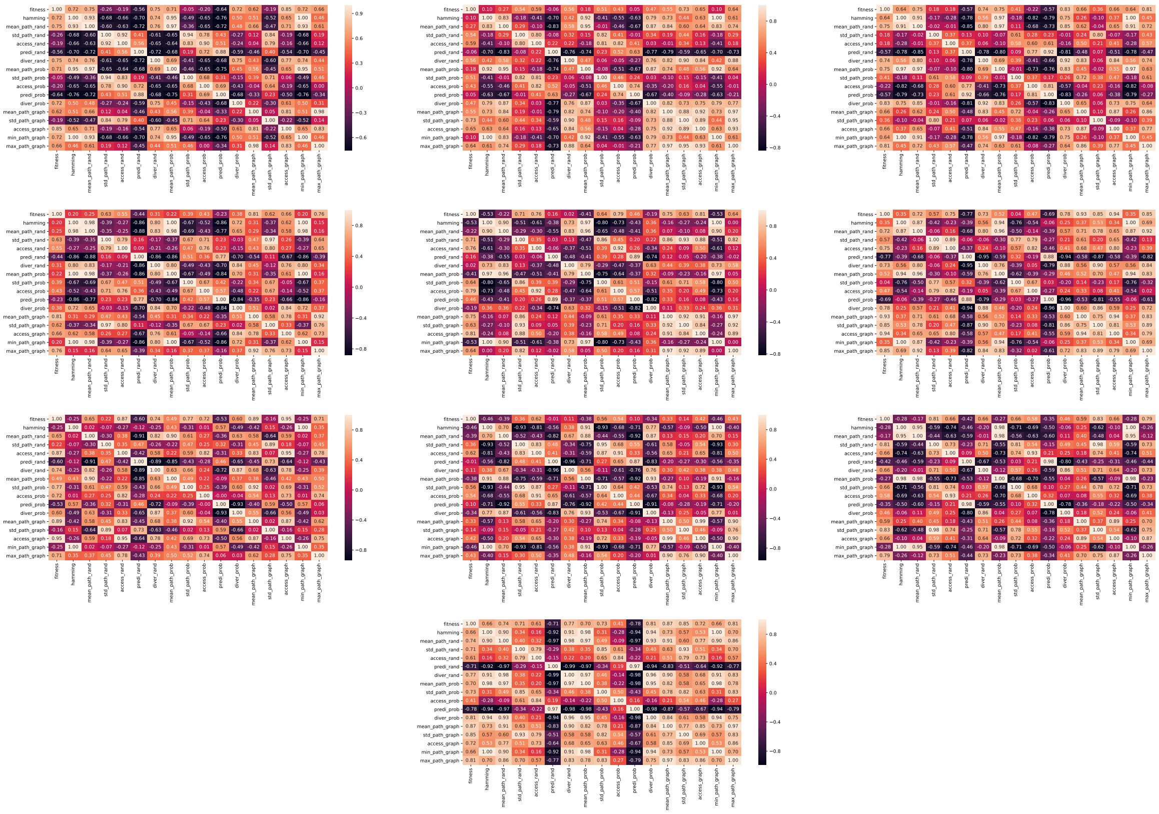

FIG. 8. Correlation tables specifying the level of correlation between pair of variables for the NK fitness landscape. The abbreviations of the variables in the label are declared in Sec. 2.3. The parameter values are $N = 8$ and $K = 3$.

[1] N. C. Wu, L. Dai, C. A. Olson, J. O. Lloyd-Smith, and R. Sun, Adaptation in protein fitness landscapes is facilitated by indirect paths, Elife **5**, e16965 (2016).
